# GHSR and TLR4 collectively promote hepatic inflammation and aberrant repair in alveolar echinococcosis

**DOI:** 10.3389/fimmu.2026.1919853

**Published:** 2026-09-08

**Authors:** Guangfeng Chen, Tanfang Zhou, Xia Chen, Ayinula Tuohetali, Ya Song, Mutailipu Maimaiti, Kalibixiati Aimulajiang, Jiang Zhu

**Affiliations:** 1 The First Affiliated Hospital of Shihezi University, Shihezi, China; 2 Department of Abdominal Surgery, The Third People Hospital of Xinjiang Uygur Autonomous Region, Urumqi, China; 3 State Key Laboratory of Pathogenesis, Prevention and Treatment of High Incidence Diseases in Central Asia, Clinical Medicine Institute, The First Affiliated Hospital of Xinjiang Medical University, Urumqi, China; 4 College of Veterinary Medicine, Xinjiang Agricultural University, Urumqi, China

**Keywords:** Alveolar echinococcosis, *E. multilocularis*, GHSR, inflammation, liver, TLR4

## Abstract

**Background and objective:**

Alveolar echinococcosis (AE) is a fatal zoonotic parasitic disease that causes progressive hepatic injury through chronic inflammation, aberrant proliferative repair, and liver fibrosis. GHSR and TLR4 individually regulate hepatic inflammation and proliferation; however, their synergistic roles in *Echinococcus multilocularis* (*E. multilocularis*)-induced AE remain unclear.

**Methods:**

Wild-type (WT), GHSR-knockout (GHSR^-/-^), and TLR4-knockout (TLR4^-/-^) murine AE models were established, and comprehensive evaluations of hepatic pathology, cell proliferation, macrophage polarization, inflammatory factors, and hepatic proteomic profiles were performed at 12 weeks post-infection.

**Results:**

Knockout of either GHSR or TLR4 alleviated AE-induced hepatic lesions, fibrosis, and liver dysfunction. Mechanistically, their deletion suppressed PI3K/Akt-mediated proliferation and TLR4/MyD88/NF-κB-mediated inflammation, reduced M1/M2 macrophage infiltration, decreased pro-inflammatory cytokines (IL-6, TNF-α, and TGF-β1), and elevated anti-inflammatory IL-10. A positive expression correlation between GHSR and TLR4 was confirmed in AE, Proteomics further verified their coordinated modulation of hepatic inflammation and aberrant repair.

**Conclusions:**

Collectively, upregulated GHSR and TLR4 jointly promote Echinococcus multilocularis-associated hepatic damage via sustaining persistent inflammation and dysregulated proliferative tissue repair. This study demonstrates coordinated pro-pathological involvement of GHSR and TLR4 in AE progression and provides *in vivo* and proteomic evidence supporting the exploration of targeted interventions against parasitic liver disorders.

## 1 Introduction

Alveolar echinococcosis (AE) is a severe, chronic, and fatal parasitic liver disease caused by larval infection with *Echinococcus multilocularis* (*E. multilocularis*). Characterized by invasive, tumor-like proliferation and progressive hepatic destruction, AE is clinically recognized as “parasitic liver cancer” (1). Its long latent period and atypical early symptoms often delay clinical diagnosis, resulting in irreversible liver damage and limited surgical options in most advanced cases (2). Currently, albendazole (ABZ) is the first-line anti-parasitic drug for AE; however, its clinical efficacy is severely limited by low bioavailability, prolonged treatment dependence, and potential drug resistance. Due to insufficient understanding of the regulatory mechanisms underlying AE-related hepatic growth remodeling and immune dysregulation, effective therapeutic targets and standardized intervention strategies remain lacking (3, 4). Therefore, elucidating the key molecular regulators governing hepatic inflammatory injury and aberrant proliferative repair during *E. multilocularis* infection is essential for advancing the pathological understanding of AE and identifying novel therapeutic targets.

Growth hormone secretagogue receptor (GHSR), a G protein-coupled receptor (GPCR), modulates growth hormone secretion, appetite regulation, and energy metabolism. It promotes cell proliferation via the PI3K/Akt signaling pathway and exerts complex roles in immune responses (5, 6). Studies have revealed that GHSR exhibits a potential double-edged sword effect in the liver (7, 8). On one hand, binding of GHSR to its ligand ghrelin inhibits activation of the TLR4/NF-κB inflammatory pathway (9, 10) and mediates macrophage polarization toward the M2 phenotype, thereby exerting an anti-inflammatory effect and alleviating liver injury (11). On the other hand, activation of GHSR itself can promote M1 macrophage polarization and exert pro-inflammatory effects (12–15). In addition, GHSR activation upregulates cyclin expression (e.g., Cyclin D1) and facilitates the transition of hepatocytes from the G0/G1 phase to the S phase of the cell cycle, thereby promoting hepatocellular proliferation (16–18). Recent studies have demonstrated that inhibition (19, 20) or knockout (13–15, 21) of the GHSR gene suppresses hepatocellular proliferative repair, inhibits inflammatory pathway activation, promotes M2 macrophage polarization, and significantly attenuates hepatic inflammatory injury. However, the phenotypic characteristics and pathological implications of GHSR during *E. multilocularis*-induced hepatic injury remain poorly defined.

Toll-like receptor 4 (TLR4), a pivotal pattern recognition receptor (PRR), plays a critical role in pathogen recognition, innate immune activation, inflammatory response promotion, and immune regulation. Activation of TLR4 triggers myeloid differentiation primary response 88 (MyD88)-dependent and TIR-domain-containing adapter-inducing interferon-β (TRIF)-dependent signaling pathways, which promote intracellular activation of the nuclear factor-κB (NF-κB) pathway and regulate M1 macrophage polarization alongside the secretion of pro-inflammatory cytokines including tumor necrosis factor-α (TNF-α) and interleukin-6 (IL-6) (22–24). Current studies have demonstrated that in chronic liver diseases, including non-alcoholic fatty liver disease (NAFLD) (25, 26), hepatitis (27, 28), hepatic fibrosis (29), liver cirrhosis (30, 31) and hepatocellular carcinoma (HCC) (32–34), high expression of TLR4 activates inflammatory pathways, further exacerbating hepatic inflammatory injury and disease progression. Conversely, TLR4 inhibition (35–37) or gene knockout (38, 39) exerts the opposite biological effect by alleviating hepatic inflammatory injury. However, the regulatory role of TLR4 in hepatic AE has not been fully elucidated to date; it may influence AE progression by modulating immune-inflammatory responses and cell proliferation/apoptosis (40–42). Notably, emerging evidence (43) potential functional association between the TLR4 cascade and the ghrelin/GHSR pathway in parasitic liver injury, providing a preliminary basis for exploring their functional correlation during AE pathogenesis. Nevertheless, the cooperative pathological roles of TLR4 and GHSR in *E. multilocularis*-induced liver damage remain unclear.

To date, most studies have only explored the individual roles of GHSR or TLR4 in chronic liver diseases, while their coordinated expression pattern and cooperative pathological functions during AE progression remain poorly elucidated. In this study, wild-type, GHSR-knockout, and TLR4-knockout murine AE models were established to comparatively analyze hepatic pathological lesions, collagen deposition, cell proliferation, macrophage polarization, inflammatory cytokine profiles, and global proteomic changes. This study aimed to characterize the correlated expression of GHSR and TLR4 and clarify their coordinated contributions on immune-inflammatory imbalance and aberrant hepatic proliferative repair upon *E. multilocularis* infection. Our findings provide systematic *in vivo* phenotypic and proteomic evidence for the cooperative pro-pathological roles of GHSR and TLR4, offering novel insights into AE pathogenesis and potential therapeutic targets for parasitic liver diseases.

## 2 Materials and methods

### 2.1 Animals

All wild-type (WT), GHSR-/- (Cat. NO. NM-KO-00007) and TLR4-/- (Cat. NO. NM-KO-18052) female C57BL/6 mice used in this study were provided by Shanghai Southern Model Biotechnology Co., Ltd. The mice were 8–10 weeks old with a body weight of 22 ± 4 g. All mice were housed in a specific pathogen-free (SPF) barrier environment at the Animal Experiment Center of Xinjiang Medical University under the conditions of constant temperature and humidity with a 12* h* light/dark cycle, and had free access to standard chow and sterile water. For 24 h before the experiment, mice were fasted while sterile water remained available ad libitum. This study was approved by the Institutional Animal Care and Use Committee (IACUC) of the First Affiliated Hospital of Xinjiang Medical University (Approval No.: IACUC-20230321-14), and all experimental procedures were strictly performed in accordance with its established guidelines.

### 2.2 Hepatic *E. multilocularis* infection model preparation and experimental design

Protoscoleces (PSCs, the infectious larval stage of *E. multilocularis*) were isolated from parasite vesicles of infected gerbils at 24 weeks post-infection under aseptic conditions. Briefly, vesicular fluid and germinal layer tissues were collected, and PSCs were purified via mechanical trituration, repeated sterile PBS washing, and sterile filtration. The purified PSCs were further digested with 1% pepsin (pH 3.0) at 37 °C for 30 min. Eosin staining confirmed a PSC viability higher than 95%, and only high-viability PSCs were used for subsequent infection.

For mouse model establishment, mice were deeply anesthetized via inhalation of 3% isoflurane (EZVET, Wuhan, China) mixed with oxygen. Adequate anesthetic depth was confirmed by the loss of the pedal withdrawal reflex, after which a laparotomy was performed to expose the hepatic portal vein. A total of 4000 viable PSCs suspended in 100 µL sterile 0.9% normal saline were injected into the hepatic portal vein (44). Mice resumed normal feeding 12 h after surgery.

Mice were randomly divided into three groups (six mice per group): WT infection group, GHSR^-/-^ infection group and TLR4^-/-^ infection group. To eliminate potential cage effects, mice from different groups were randomly allocated to multiple cages throughout the experiment. All animal treatments, sample collection and data analyses were performed by blinded investigators. At 12 weeks post-infection of feeding, all mice were re-anesthetized with 3% inhaled isoflurane prior to euthanasia via rapid cervical dislocation. Complete death was verified by persistent absence of spontaneous respiration, heartbeat and pain reflexes for no less than 3 minutes. Subsequently, body weight and liver weight were recorded, and serum as well as liver tissue samples were collected for downstream experiments.

### 2.3 Histopathological analysis

Fresh liver tissue specimens were fixed in 4% paraformaldehyde (Biosharp, Hefei, China), embedded in paraffin, and cut into 3 μm continuous sections. After deparaffinization and gradient hydration, the sections were subjected to hematoxylin-eosin (H&E) staining, Masson’s trichrome staining and periodic acid-Schiff (PAS) staining (Solarbio, Beijing, China). H&E staining was used to evaluate the morphological alterations and lesion scope of liver tissues. Masson’s trichrome staining was applied to quantify collagen fiber distribution, while PAS staining was performed to analyze the localization of glycogen in hepatocytes.

All stained sections were observed and photographed under an Olympus BX43 light microscope (Olympus, Tokyo, Japan). ImageJ v1.8.0 software (National Institutes of Health, USA) was used for quantitative image analysis. The relative area of lesions and collagen fibers was calculated as the ratio of the lesion area or Masson-positive blue-stained area to the total area of liver sections, respectively. Each group contained six biological replicates (n = 6).

### 2.4 Immunohistochemistry

After dewaxing and gradient dehydration, liver tissue sections were subjected to heat-induced antigen retrieval using citrate buffer (pH 6.0) and Tris-EDTA buffer (Thermo Fisher Scientific, MA, USA), respectively, in accordance with the manufacturers’ instructions. Subsequently, endogenous peroxidase activity was blocked with 3% hydrogen peroxide solution to reduce non-specific staining. The slides were then blocked with goat serum solution (Proteintech, Wuhan, China) for 30 min at room temperature. Primary antibody incubation was performed overnight at 4 °C; detailed information including source and working dilution of all primary antibodies is summarized in **Table 1**. Thereafter, the sections were incubated with HRP-conjugated goat anti-rabbit or anti-mouse secondary antibody (Proteintech, Wuhan, China) for 1 h at room temperature. Color development was achieved using a DAB kit (Proteintech, Wuhan, China), followed by hematoxylin counterstaining (BIGB-Bio, Beijing, China) and slide mounting. All stained specimens were visualized under an Olympus BX43 light microscope (Olympus, Tokyo, Japan), and quantitative analysis of positive staining area was conducted with ImageJ v1.8.0 software (National Institutes of Health, USA).

**Table 1:** Source and dilution information of antibodies for IHC and WB detection.

Target protein	Host species	Catalog No.	Manufacturer	Dilution
Ghrelin	Rabbit	ab209790	Abcam (Cambridge, UK)	IHC:1/8000
p-PI3K p110β (Ser1070)	Rabbit	bs-6417R	Bioss Inc. (Woburn, MA, USA)	IHC:1/300 WB:1/2000
p-Akt (Ser473)	Mouse	66444-1-Ig	Proteintech Group (Wuhan, China)	IHC:1/200 WB:1/3000
PI3K p110β	Rabbit	20584-1-AP	Proteintech Group (Wuhan, China)	WB:1/1000
Akt	Rabbit	10176-2-AP	Proteintech Group (Wuhan, China)	WB:1/4000
Ki67	Rabbit	AF0198	Affinity Biosciences (Ohio, USA)	IHC:1/200
PCNA	Rabbit	10205-2-AP	Proteintech Group (Wuhan, China)	IHC:1/4000
Cyclin D1	Rabbit	ab134175	Abcam (Cambridge, UK)	IHC:1/100 WB:1/10000
Cyclin E1	Rabbit	11554-1-AP	Proteintech Group (Wuhan, China)	IHC:1/500 WB:1/1000
GHSR	Rabbit	30602-1-AP	Proteintech Group (Wuhan, China)	IHC:1/300 WB:1/1000
TLR4	Rabbit	19811-1-AP	Proteintech Group (Wuhan, China)	IHC:1/300 WB:1/1000
MyD88	Rabbit	AF5195	Affinity Biosciences (Ohio, USA)	IHC:1/150 WB:1/1000
NF-κB p65	Rabbit	AF5006	Affinity Biosciences (Ohio, USA)	IHC:1/150 WB:1/1000
CD68	Rabbit	ab283654	Abcam (Cambridge, UK)	IHC:1/100
iNOS	Rabbit	ab283655	Abcam (Cambridge, UK)	IHC:1/2000 WB:1/2000
Arg-1	Rabbit	16001-1-AP	Proteintech Group (Wuhan, China)	IHC:1/300 WB:1/5000
β-Actin	Rabbit	20536-1-AP	Proteintech Group (Wuhan, China)	WB:1/4000

### 2.5 Multiplex fluorescent immunohistochemistry

First, tissue sections were dewaxed in xylene and rehydrated using a graded ethanol series. Antigen retrieval was then conducted at high temperature with either Tris-EDTA buffer (pH 9.0) or sodium citrate buffer (pH 6.0) to expose antigenic epitopes. Endogenous peroxidase activity was blocked by incubation with 3% hydrogen peroxide solution. The sections were incubated overnight at 4 °C with primary antibodies, including rabbit anti-GHSR (1:200, 30602-1-AP, Proteintech), mouse anti-CD68 (1:200, 66231-2-Ig, Proteintech), mouse anti-α-SMA (1:400, A2547, Sigma), mouse anti-TLR4 (1:200, 66350-1-Ig, Proteintech), rabbit anti-MyD88 (1:200, AF5195, Affinity), and rabbit anti-NF-κB p65 (1:200, AF5006, Affinity). On the next day, the sections were rinsed with PBS and incubated with secondary antibodies for 50 min at room temperature. Tyramide signal amplification (TSA) reagents (Servicebio, Wuhan, China) were applied to enhance fluorescence signals. For multiplex immunofluorescence staining, antigen retrieval and blocking procedures were repeated as described above. Cell nuclei were counterstained with DAPI (Servicebio, Wuhan, China). All sections were mounted with neutral balsam to avoid drying and contamination, and observed under a fluorescence microscope (Nikon, Dongguan, Japan) for subsequent quantitative analysis.

Fluorescence colocalization was analyzed using Image-Pro Plus 6.0 software (Media Cybernetics, USA). Representative fields of liver tissue were randomly captured, and background fluorescence was subtracted before quantification. Pearson’s correlation coefficient (*r*, range: −1 to 1) was used to assess the spatial linear correlation between two fluorescent signals; values closer to 1 indicate stronger spatial consistency. The overlap coefficient (*R*, range: 0 to 1) was used to calculate the area overlap ratio of valid fluorescent signals, with a value of 1 representing complete overlap. Colocalization degrees were classified according to commonly used thresholds. High-level colocalization was defined as *r* ≥ 0.8 and *R* ≥ 0.8, whereas low-level colocalization was defined as 0.3 ≤ *r* < 0.8 and 0.2 ≤ *R* < 0.8. All experiments were performed with three biological replicates per group (n = 3).

### 2.6 Western blotting

Total proteins were extracted from mouse liver tissues using RIPA lysis buffer supplemented with protease and phosphatase inhibitor cocktails. Tissues were homogenized on ice and centrifuged to harvest supernatants. Protein concentrations were quantified with the BCA Protein Assay Kit (Solarbio, Beijing, China). Proteins were separated via SDS-PAGE (Biotides, Beijing, China) and electrotransferred onto PVDF membranes (Sigma-Aldrich, Shanghai, China). Membranes were blocked with 5% non-fat milk (Wako, Guangzhou, China), followed by overnight incubation with primary antibodies at 4 °C; detailed information of primary antibodies including catalog numbers, suppliers and working dilutions is provided in **Table 1**. After PBS washing, blots were incubated with HRP-conjugated secondary antibody (multi-rAb HRP-goat anti-rabbit, 1:5000, RGAR001, Proteintech, Wuhan, China) for 1 h at room temperature. Target protein bands were detected using an ECL chemiluminescence kit (Biosharp, Hefei, China).Band gray values were quantified with ImageJ v1.8.0 software (National Institutes of Health, Bethesda, MD, USA).

### 2.7 Quantitative real-time polymerase chain reaction

Total RNA was extracted from mouse liver tissues using Trizol reagent (Solarbio, Beijing, China), and the RNA concentration was quantified. Subsequently, reverse transcription was performed with the HiScript III RT SuperMix for qPCR Kit (Vazyme, Nanjing, China), and quantitative real-time polymerase chain reaction (qRT-PCR) was carried out using the SYBR Green I Detection Kit (Vazyme, Nanjing, China). The entire detection process was monitored and analyzed on a Bio-Rad Real-Time PCR System (Bio-Rad, Foster City, CA, USA). All primers were designed and synthesized by Sangon Biotech (Shanghai, China), with all primer sequences provided in **Table 2**.

**Table 2:** Primers and conditions used in qRT-PCR reactions.

Primer name	Primer sequences		Length (bp)
Ghrelin	Forward	GGACCTCCTCAGGGGACCAGAT	22
	Reverse	GGTCGGGGTAGCGGCTGAA	19
GHSR	Forward	ACCGTGATGGTATGGGTGTCG	21
	Reverse	CACAGTGAGGCAGAAGACCG	20
Cyclin D1	Forward	GCTGCGAAGTGGAAACCATC	20
	Reverse	CCTCCTTCTGCACACATTTGAA	22
Cyclin E1	Forward	CGGTATATGGCGACACAAGA	20
	Reverse	ACATACGCAAACTGGTGCAA	20
TLR4	Forward	GCTATCTGTGAGCGTGTAT	19
	Reverse	ACGGCAACTTGGACCTG	17
MyD88	Forward	GCAGAACCAGGAGTCCGAGAAG	22
	Reverse	GATGCCTCCCAGTTCCTTTGTTTG	24
NF-κB p65	Forward	ATGGGAAACCGTATGAGCCTGTG	23
	Reverse	AGTTGTAGCCTCGTGTCTTCTGTC	24
iNOS	Forward	ACATCGACCCGTCCACAGTAT	21
	Reverse	CAGAGGGGTAGGCTTGTCTC	20
Arg-1	Forward	CTCCAAGCCAAAGTCCTTAGAG	22
	Reverse	GGAGCTGTCATTAGGGACATCA	22
IL-2	Forward	TGGAGGAGGAGGAGGAGGAG	20
	Reverse	GAGGAGGAGGAGGAGGAGGA	20
IL-6	Forward	TTCTTGGGACTGATGCTGGTG	21
	Reverse	CACAACTCTTTTCTCATTTCCACGA	25
TNF-α	Forward	CAGGCGGTGCCTATGTCTC	19
	Reverse	CGATCACCCCGAAGTTCAGTAG	22
TGF-β1	Forward	TGATACGCCTGAGTGGCTGTCT	22
	Reverse	CACAAGAGCAGTGAGCGCTGAA	22
IL-10	Forward	GCCACGGCACAGTCATTGA	19
	Reverse	TGCTGATGGCCTGATTGTCTT	21
β-Actin	Forward	GGGACGACATGGAGAAGA	18
	Reverse	ACGACCAGAGGCATACAG	18

### 2.8 Enzyme-linked immunosorbent assay

The levels of Ghrelin, IL-2, IL-6, TNF-α, TGF-β1 and IL-10 in mouse serum and liver tissues were quantitatively detected using ELISA kits (Lapuda Biotechnology, Nanjing, China) in accordance with the manufacturer’s instructions. The absorbance values were measured at 450 nm using a BioTek microplate reader (BioTek, Vermont, USA).

### 2.9 EdU assay

Before tissue collection, mice were subjected to *in vivo* EdU labeling by intraperitoneal injection of 5-ethynyl-2′-deoxyuridine (EdU) at a dose of 50 mg/kg body weight, 2 hours prior to euthanasia. Liver tissues were then harvested, fixed, and embedded for sectioning. After dewaxing and gradient dehydration, sections were processed using the BeyoClick™ EdU-555 Cell Proliferation Assay Kit (Beyotime, Shanghai, China) according to the manufacturer’s instructions. Briefly, freshly prepared Click reaction buffer was applied to cover the tissue sections, followed by incubation for 30 minutes at room temperature in the dark. Sections were then washed three times with PBS containing 3% BSA (absin, Wuhan, China), 3–5 minutes each wash. Nuclear counterstaining was performed with 1 × Hoechst 33342 (Beyotime, Shanghai, China) for 10 minutes at room temperature in the dark. Fluorescence images were captured using a fluorescence microscope (Nikon, Tokyo, Japan).

### 2.10 Proteomic sequencing analysis

Five liver samples from WT, GHSR^-^/^-^ and TLR4^-^/^-^ groups were subjected to label-free quantitative (LFQ) proteomic analysis. Samples were rinsed with pre-chilled phosphate-buffered saline (PBS), ground into powder under liquid nitrogen, and lysed with radioimmunoprecipitation assay (RIPA) buffer containing complete protease and phosphatase inhibitor cocktails, followed by sonication. Supernatants were collected after centrifugation, and total protein concentrations were quantified using the bicinchoninic acid (BCA) assay. Total proteins were digested with trypsin (1:50, w/w). Peptides were separated on an EASY-nLC 1200 ultra-high-performance liquid chromatography (UHPLC) system, then analyzed on an Orbitrap Exploris™ 480 mass spectrometer operating in data-dependent acquisition (DDA) mode. Raw data were processed in MaxQuant software with LFQ normalization enabled.Differentially expressed proteins (DEPs) were defined by |log_2_(fold change)| ≥ 1 and *p* < 0.05, consistent with the thresholds shown in the volcano plot. From these DEPs, inflammation-related proteins were further filtered for heatmap visualization and functional enrichment. Gene Ontology (GO) and Kyoto Encyclopedia of Genes and Genomes (KEGG) pathway enrichment analyses were performed to annotate biological functions.

### 2.11 Statistical analysis

All data were analyzed using GraphPad Prism 10.1.2 (GraphPad Software, San Diego, CA, USA). Quantitative data are presented as means ± standard deviation (SD). The Shapiro-Wilk test was used to evaluate data normality. For normally distributed data, unpaired Student’s t-test was applied for two-group comparisons, and two-way ANOVA followed by Šidák’s post-hoc multiple-comparisons test was used for multigroup comparisons of multiple related indicators. For datasets that did not meet the normality assumption, the non-parametric Mann–Whitney U test was used for two-group analysis. The number of biological replicates (n) varies among different experiments, with n = 3, 5, or 6 independent mice per group; the exact n value for each panel is explicitly indicated in the corresponding figure legend. All quantitative analyses in this study were performed using the statistical methods described above. Statistical significance was defined as follows: *P < 0.05, **P < 0.01, ***P < 0.001, ****P < 0.0001.

## 3 Results

### 3.1 GHSR deficiency attenuates hepatic injury during AE

**Figure 1:**
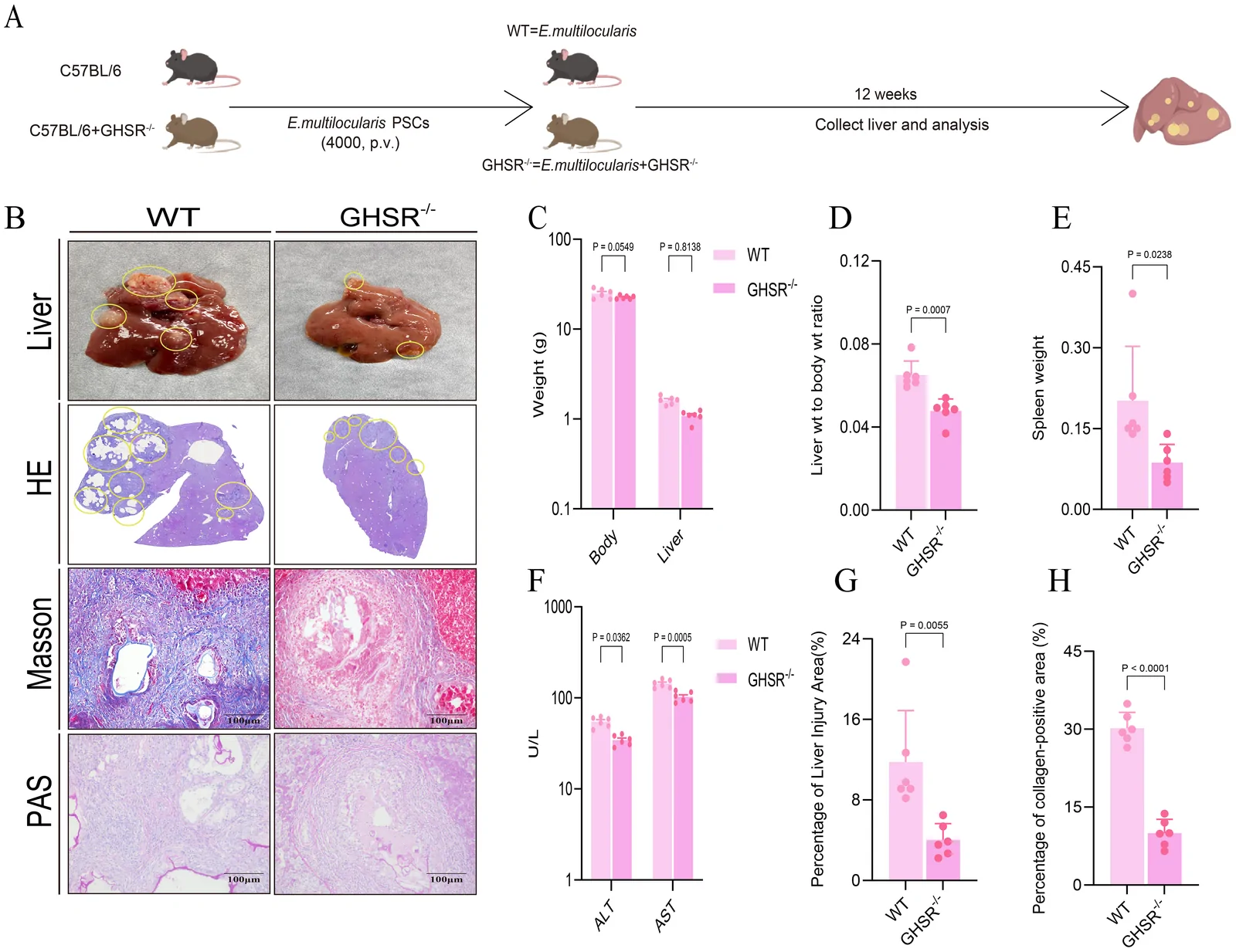
GHSR deficiency attenuates hepatic injury during AE. **(A)** Schematic diagram of animal infection experimental design. **(B)** Gross morphology of liver tissues, together with representative H&E, Masson,s trichrome and PAS staining of hepatic paraffin sections. **(C)** Body weight and liver weight of experimental animals. **(D)** Liver-to-body weight ratio. **(E)** Spleen weight. **(F)** Serum ALT and AST activities reflecting liver injury. **(G)** Quantitative statistics of hepatic lesion area from H&E staining. **(H)** Quantitative analysis of collagen deposition positive area based on Masson staining. Y-axis in **(C, F)** is plotted on a logarithmic scale to accommodate wide data ranges. (n = 6). Statistical significance: *P < 0.05, **P < 0.01, ***P < 0.001, ****P < 0.0001.

A murine model of hepatic *E. multilocularis* infection was successfully established in WT and GHSR-/- mice via intrahepatic portal vein inoculation, and all analyses were performed at 12 weeks post-infection (**Figure 1A**). Gross examination revealed a markedly smaller hepatic lesion area in GHSR-/- mice than those in WT controls. Histopathological analyses further demonstrated reduced lesion burden, collagen deposition, and glycogen accumulation in the livers of GHSR-deficient mice (**Figures 1B, G, H**).

Although body weight and absolute liver weight did not differ significantly between groups (**Figure 1C**), GHSR-/- mice exhibited lower liver-to-body weight ratios and reduced spleen weights compared with WT mice (**Figures 1D, E**). Consistent with the histological findings, serum biochemical analyses showed lower ALT and AST levels in GHSR-/- mice, indicating attenuated hepatic injury and preserved liver function (**Figure 1F**). Collectively, these findings demonstrate that GHSR deficiency alleviates parasite-induced hepatic injury, fibrosis, and hepatosplenic enlargement during AE, thereby limiting disease progression.

### 3.2 GHSR exhibits spatial association with TLR4-related inflammatory and reparative signaling in infected livers

**Figure 2:**
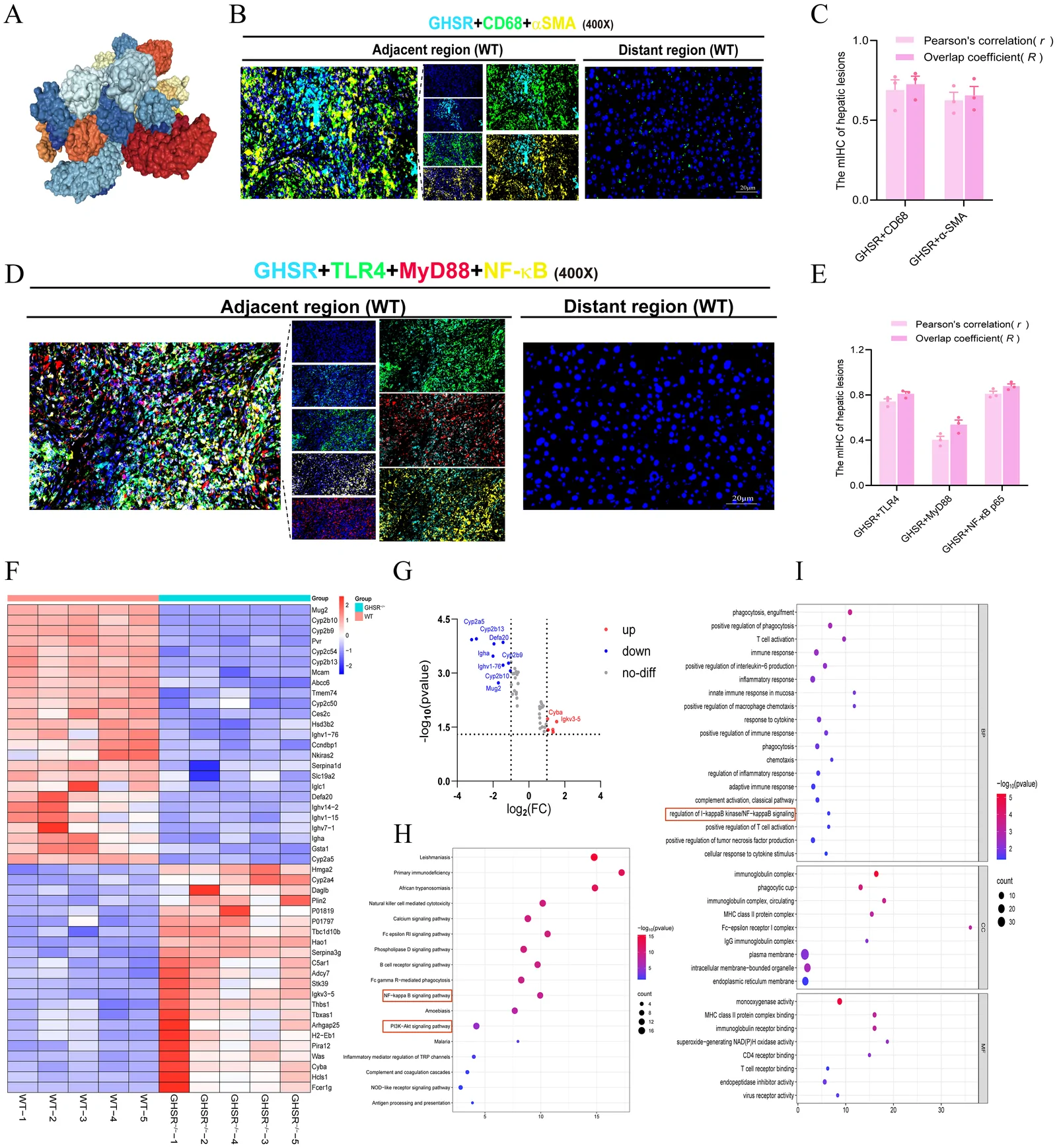
GHSR exhibits spatial association with TLR4-related inflammatory and reparative signaling in infected livers. **(A)** Molecular docking analysis of the physical interaction between GHSR and TLR4. **(B, C)** Representative multiplex mIHC images and quantitative colocalization analysis for GHSR (blue), CD68 (green) and α-SMA (yellow) in perilesional and distant hepatic regions of *E. multilocularis*-infected WT mice. Colocalization was quantified by Pearson’s correlation coefficient (*r*) and overlap coefficient (*R*) (n = 3). **(D, E)** Representative mIHC images and quantitative colocalization statistics of GHSR (blue), TLR4 (green), MyD88 (red) and NF-κB p65 (yellow) in perilesional and distant liver areas of infected WT mice. Pearson’s r and overlap R were calculated to evaluate spatial colocalization (n = 6). **(F)** Heatmap of DEPs after Z-score normalization across WT and GHSR-/- groups. Red denotes elevated expression, blue denotes reduced expression (n = 5). **(G)** Volcano plot illustrating global protein fold changes. X-axis: log_2_(fold change); Y-axis: −log_10_(*P*-value). Cutoff criteria: |log_2_FC| ≥ 1 and *P* < 0.05. Red = significantly upregulated proteins, blue = significantly downregulated proteins, gray = no significant change; key candidate proteins are annotated (n = 5). **(H)** KEGG pathway enrichment analysis based on identified DEPs. Dot size corresponds to enriched gene count, color gradient represents −log_10_(*P*-value) (n = 5). **(I)** GO functional enrichment analysis of DEPs, with dot size and color coded consistent with **(G)** (n = 5). Statistical significance: *P < 0.05, **P < 0.01, ***P < 0.001, ****P < 0.0001.

To explore the potential structural association between GHSR and TLR4, protein-protein molecular docking was performed. Ten predicted complex models were generated, and Model 1, which exhibited the optimal docking score (−250.96, confidence score = 0.8828), was selected for further analysis. A distinct predicted binding interface was observed between GHSR (PDB ID: 3T6Q) and TLR4 (PDB ID: 1ZI8). Notably, this in silico prediction only suggests a potential structural association and cannot confirm physical or functional interaction between these two molecules (**Figure 2A**).

To characterize the signaling networks associated with GHSR during *E. multilocularis* infection, mIHC was performed to examine the spatial distribution of key inflammatory and reparative molecules in perilesional hepatic tissues. GHSR exhibited overlapping localization with the macrophage marker CD68 and the fibrotic marker α-SMA (**Figure 2B**), and was also detected in regions enriched for components of the TLR4/MyD88/NF-κB signaling pathway (**Figure 2D**). Quantitative analyses further confirmed the spatial association of GHSR with these inflammatory and reparative markers (**Figures 2C, E**).

To further investigate GHSR-associated molecular alterations, LFQ LC–MS/MS proteomic profiling was performed using liver tissues from WT and GHSR-/- mice. A total of 185 DEPs were identified, among which 47 inflammation-related proteins were selected for downstream analyses (**Figure 2F**). Within this subset, six proteins showed increased abundance, while nine proteins exhibited decreased abundance following GHSR deletion (**Figure 2G**, **Table 3**).

**Table 3:** The most significantly differentially expressed proteins between the WT group and GHSR^-/-^ group.

Protein IDs	Protein name	Fold change	*P* value	Regulation
A0A0G2JFE9	Ighv1-76	1.4426	0.0006	Down
Q91X75	Cyp2a5	3.1975	0.0001	Down
P12791	Cyp2b10	2.7158	0.0004	Down
P28666	Mug2	1.7064	0.0019	Down
A6H6J2	Cyp2b13	2.9333	0.0001	Down
P12790	Cyp2b9	1.1348	0.0005	Down
A0A0A6YXW6	Igha	2.0049	0.0003	Down
Q45VN2	Defa20	1.9520	0.0002	Down
A0A140T8M6	Igkv3-5	1.5475	0.0223	Up
Q61462	Cyba	1.0414	0.0187	Up

KEGG enrichment analysis revealed that these DEPs were enriched in pathways involved in hepatic injury and immune regulation, including NF-κB signaling, PI3K/Akt signaling, and FcγR-mediated phagocytosis (**Figure 2H**). GO enrichment analysis further assigned these proteins to biological processes and molecular functions related to inflammatory response regulation, modulation of IκB kinase/NF-κB signaling, macrophage chemotaxis, membrane organization, and immunoglobulin receptor binding (**Figure 2I**).

Taken together, these findings place GHSR within a network of inflammatory and reparative signaling pathways during *E. multilocularis* infection. The observed associations with macrophage-related functions, PI3K/Akt signaling, and the TLR4/NF-κB pathway provide a framework for subsequent investigation into GHSR-mediated regulation of hepatic inflammation and aberrant proliferative remodeling.

### 3.3 GHSR deficiency suppresses PI3K/Akt-driven proliferative remodeling in perilesional hepatic tissues

**Figure 3:**
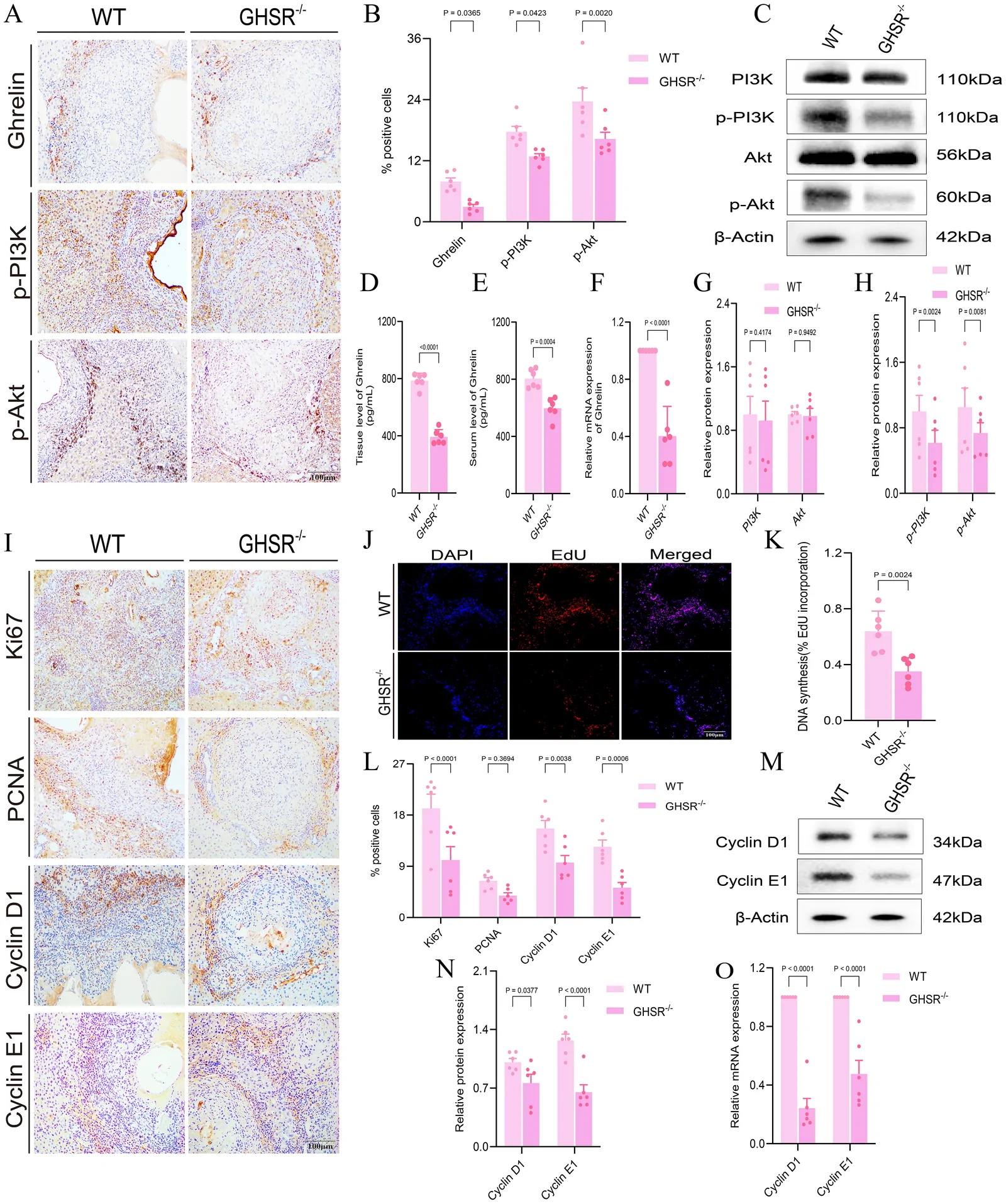
GHSR deficiency suppresses PI3K/Akt-driven proliferative remodeling in perilesional hepatic tissues. **(A, B)** Representative IHC micrographs and statistical quantification of Ghrelin, p-PI3K and p-Akt; images captured at ×200 magnification. **(C, G, H)** Western blot bands and densitometric quantification for total PI3K, Akt, phosphorylated p-PI3K, p-Akt and β-Actin. **(D, E)** ELISA quantification of Ghrelin contents in hepatic homogenate and peripheral serum. **(F)** qRT-PCR detection of hepatic Ghrelin mRNA relative expression. **(I, L)** IHC staining and positive-cell statistics of proliferation markers Ki67, PCNA, Cyclin D1 and Cyclin E1. **(J, K)** Representative EdU immunofluorescence images and quantitative analysis of peri-lesional proliferative cell proportion. **(M, N)** Western blot detection and quantification of Cyclin D1 and Cyclin E1 protein abundance. **(O)** qRT-PCR analysis of relative Cyclin D1/E1 hepatic mRNA levels. (n = 6). Statistical significance: *P < 0.05, **P < 0.01, ***P < 0.001, ****P < 0.0001.

To investigate the role of GHSR in hepatic proliferative remodeling during *E. multilocularis* infection, the expression of proliferation-related signaling molecules was examined in perilesional liver tissues. IHC analysis demonstrated reduced expression of Ghrelin, p-PI3K, and p-Akt in and around hepatic lesions of GHSR-/- mice compared with WT controls (**Figures 3A, B**). Consistent with these observations, Western blot analysis confirmed lower hepatic protein levels of p-PI3K and p-Akt in the GHSR-deficient group, whereas total PI3K and Akt expression remained unchanged (**Figures 3C, G, H**). In addition, hepatic and circulating Ghrelin levels, as well as hepatic Ghrelin mRNA expression, were reduced following GHSR deletion (**Figures 3D–F**).

EdU staining further demonstrated a lower proportion of proliferating hepatocytes in perilesional regions of GHSR-/- livers (**Figures 3J, K**). Consistently, IHC analysis revealed decreased expression of the proliferation-associated markers Ki67, PCNA, Cyclin D1, and Cyclin E1 in lesion-adjacent hepatic tissues (**Figures 3I, L**). Moreover, both protein and mRNA levels of Cyclin D1 and Cyclin E1 were lower in GHSR-deficient livers than in WT controls (**Figures 3M–O**). Collectively, these findings indicate that GHSR deficiency suppresses PI3K/Akt signaling activation and attenuates aberrant proliferative remodeling in perilesional hepatic tissues during *E. multilocularis* infection. The coordinated downregulation of cell-cycle regulators and proliferative activity suggests that GHSR drives excessive reparative responses that facilitate lesion progression.

### 3.4 GHSR deficiency remodels the hepatic inflammatory microenvironment and restrains TLR4/NF-ĸB activation

**Figure 4:**
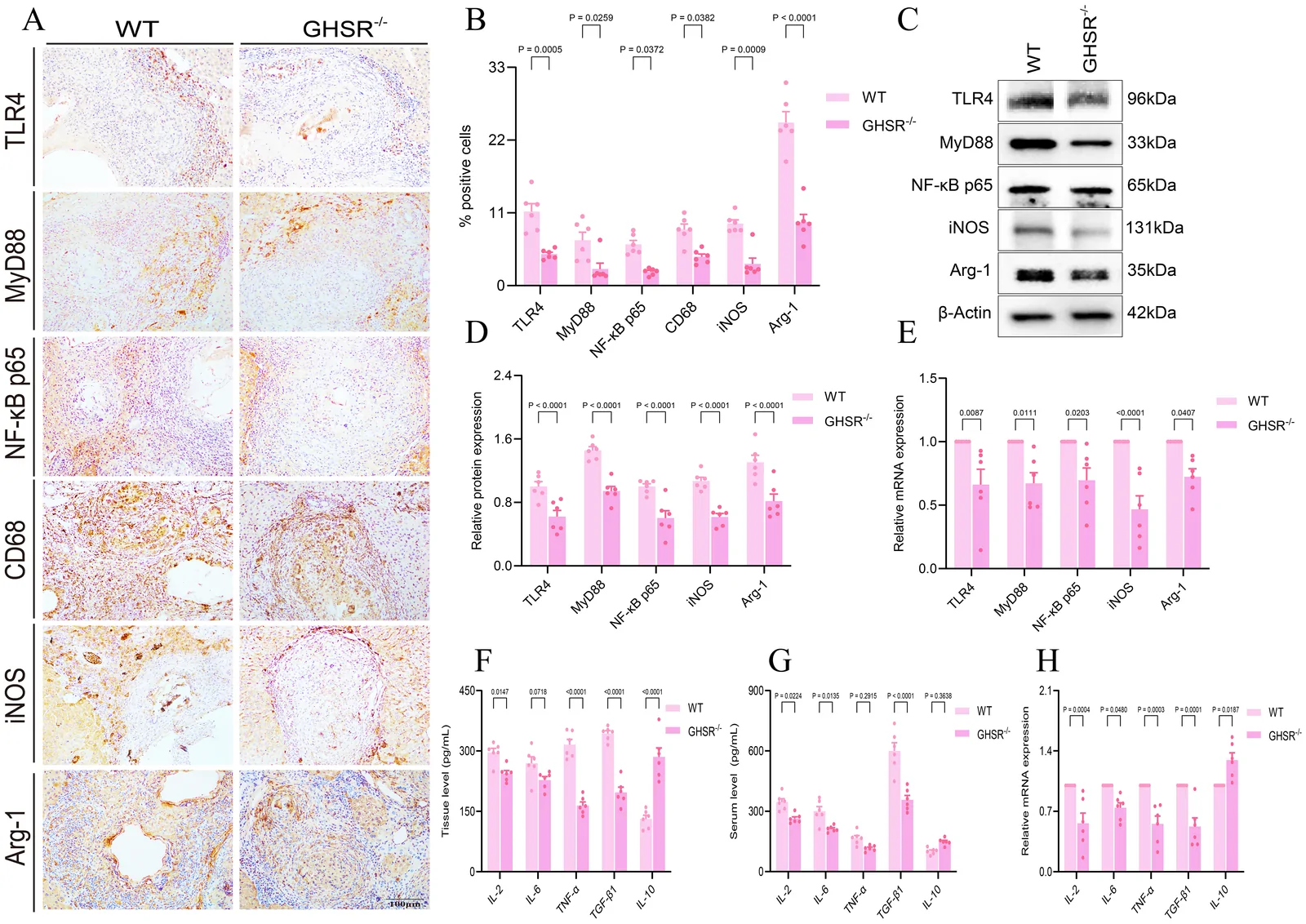
GHSR deficiency remodels the hepatic inflammatory microenvironment and restrains TLR4/NF-kB activation. **(A, B)** Representative IHC staining and quantitative statistics ofTLR4, MyD88, NF-κB p65, CD68, iNOS and Arg-1 in hepatic paraffin sections. **(C, D)** Western blot bands and densitometric quantification for TLR4, MyD88, NF-κB p65, iNOS and Arg-1; β-Actin was used as internal reference protein. **(E)** qRT-PCR detection of relative mRNA levels for the above six target genes in liver tissue. **(F, G)** ELISA detection of inflammatory cytokines (IL-2, IL-6, TNF-α, TGF-β1, IL- 10) in hepatic homogenate and peripheral serum, respectively. **(H)** qRT-PCR analysis of relative hepatic mRNA expression of IL-2, IL-6, TNF-α, TGF-β1 and IL- 10. (n = 6). Statistical significance: *P < 0.05, **P < 0.01, ***P < 0.001, ****P < 0.0001.

This study further investigated the impact of GHSR deficiency on hepatic inflammatory responses during *E. multilocularis* infection. IHC analysis demonstrated that TLR4, MyD88, NF-κB p65, CD68, iNOS, and Arg-1 were primarily localized within perilesional inflammatory regions. Most markers accumulated in the peripheral areas of inflammatory lesions, whereas Arg-1 was predominantly distributed in the central inflammatory zone (**Figure 4A**). Quantitative analysis revealed significantly decreased expression levels of these inflammatory and macrophage-associated markers in GHSR-/- mice relative to WT controls (**Figure 4B**).

Consistent with the histological findings, Western blotting and qRT-PCR analyses confirmed reduced hepatic protein and mRNA levels of TLR4, MyD88, NF-κB p65, iNOS, and Arg-1 following GHSR deletion (**Figures 4C–E**). Notably, Arg-1 expression remained relatively higher than iNOS expression across all experimental groups, suggesting the sustained M2 macrophage polarization during late-stage infection.

Furthermore, GHSR deficiency was associated with decreased secretion of pro-inflammatory cytokines (IL-2, IL-6, TNF-α, and TGF-β1) and increased IL- 10 expression in both hepatic tissues and serum (**Figures 4F–H**). Collectively, these findings indicate that GHSR deficiency suppresses TLR4/NF-κB-mediated inflammatory signaling and attenuates inflammatory mediator production during *E. multilocularis* infection. The concomitant reduction in macrophage-associated markers and pro-inflammatory cytokines suggests that GHSR contributes to the maintenance of a pro-inflammatory hepatic microenvironment, whereas its deletion alleviates parasite-induced hepatic inflammation.

### 3.5 TLR4 deficiency attenuates hepatic injury during AE

**Figure 5:**
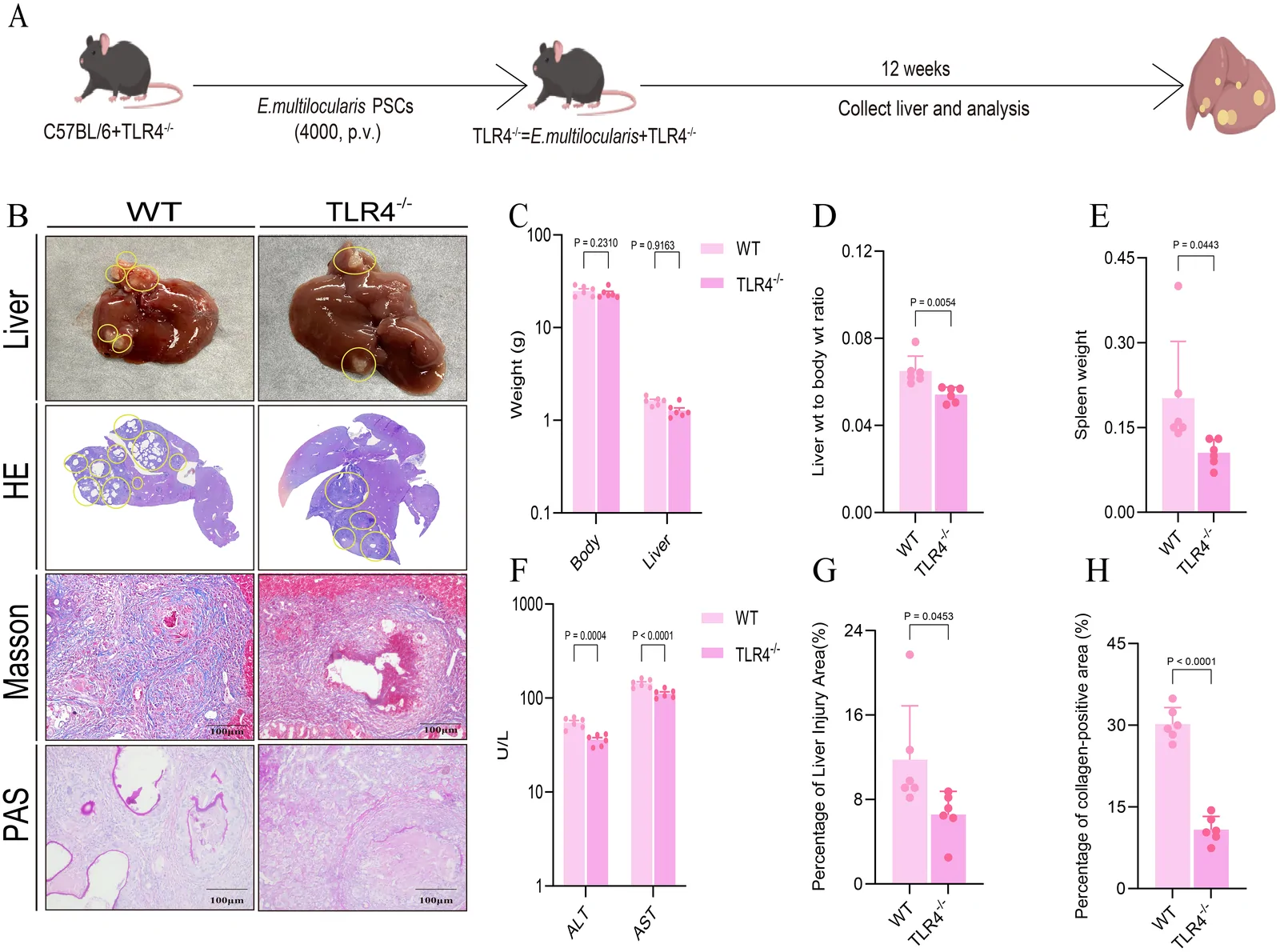
TLR4 deficiency attenuates hepatic injury during AE. **(A)** Schematic workflow for establishing TLR4-KO infected mouse model. **(B)** Gross liver morphology plus H&E, Masson and PAS staining. **(C–E)** Body/liver weight, liver/body ratio and spleen weight quantification. **(F)** Serum ALT/AST levels for liver function assessment. **(G, H)** Quantitative analysis of hepatic lesion [**(G)** H&E] and collagen deposition [**(H)** Masson]. (n = 6). Statistical significance:*P < 0.05, **P < 0.01, ***P < 0.001, ****P < 0.0001.

To determine the contribution of TLR4 to AE-associated liver injury, parallel hepatic *E. multilocularis* infection models were established in WT and TLR4-/- mice, and all analyses were performed at 12 weeks post-infection (**Figure 5A**). Macroscopic examination demonstrated a reduction in lesion burden in TLR4-deficient livers, accompanied by decreased lesion occupancy, collagen deposition, and glycogen accumulation relative to WT controls (**Figure 5B**).

Although body weight and absolute liver weight were comparable between groups (**Figure 5C**), TLR4-/- mice exhibited lower liver-to-body weight ratios and reduced spleen weights (**Figures 5D, E**), suggesting mitigated hepatosplenic pathology. Consistent with these observations, serum ALT and AST levels were reduced following TLR4 deletion, indicating preserved hepatic function (**Figure 5F**).

Histological analyses further confirmed diminished lesion expansion and collagen deposition in the livers of TLR4-/- mice (**Figures 5G, H**). Collectively, these findings demonstrate that TLR4 deficiency confers protection against parasite-triggered hepatic injury and aberrant fibrotic remodeling during AE.

### 3.6 Proteomic analyses support coordinated involvement of TLR4 in inflammatory and metabolic remodeling

**Figure 6:**
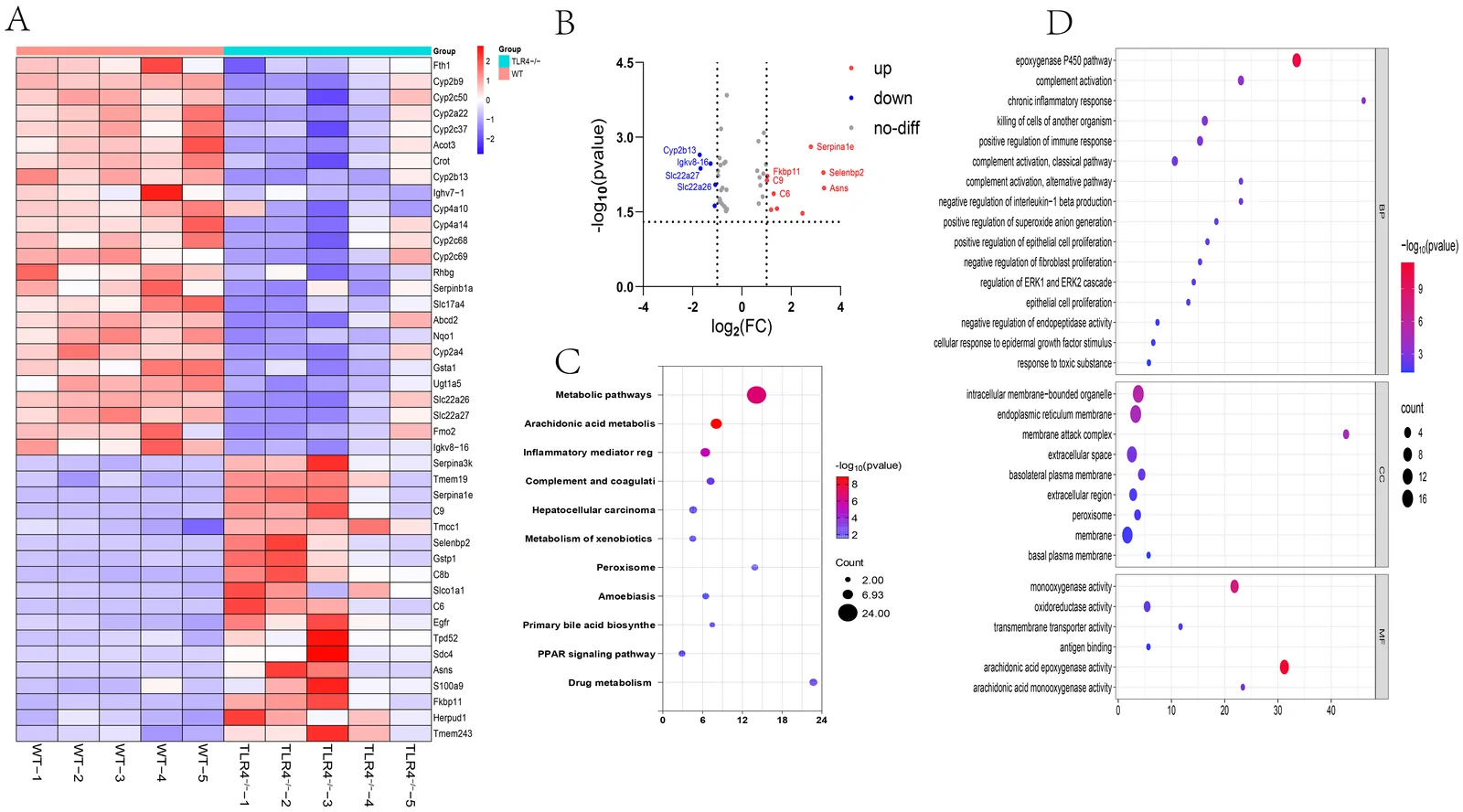
Proteomic analyses support coordinated involvement of TLR4 in inflammatory and metabolic remodeling. **(A)** Z-score normalized expression heatmap of all screened DEPs; elevated protein abundance is marked in red, while reduced expression is presented in blue. **(B)** Volcano scatter plot illustrating global protein fold changes between genotypes. Cutoff thresholds: |log2(FC)| ≥ 2 and *P* < 0.05; red = significantly upregulated DEPs, blue = significantly downregulated DEPs, gray = non-differential proteins. Representative key candidate molecules are annotated alongside corresponding dots. **(C)** Bubble plot for top enriched KEGG signaling pathways derived from DEPs; bubble size correlates with the number of enriched target proteins, and color gradient corresponds to _log1_0_(*P*-value). **(D)** GO enrichment classification partitioned into BP,CC and MF; bubble dimension stands for enriched protein count, color gradient reflects the significance of enrichment (_log1_0_(*P*-value)). (n = 5). Statistical significance: *P < 0.05, **P < 0.0l, ***P < 0.00l, ****P < 0.000l.

LFQ quantitative proteomics was performed on liver tissues from WT and TLR4^-/-^ groups to characterize TLR4-dependent molecular alterations during *E. multilocularis* infection. A total of 75 DEPs were identified, among which 43 proteins associated with inflammatory and metabolic processes were selected for downstream analyses and visualized using volcano plots and heatmaps (**Figure 6A**). Among these proteins, nine were upregulated and five were downregulated in TLR4-deficient livers relative to WT controls (**Figure 6B**, **Table 4**).

**Table 4:** The most significantly differentially expressed proteins between the WT group and TLR4^-/-^ group.

Protein IDs	Protein name	Fold change	*P* value	Regulation
A6H6J2	Cyp2b13	1.7120	0.0023	Down
A0A0G2JDG9	Igkv8-16	1.2725	0.0034	Down
Q76M72	Slc22a27	1.6794	0.0042	Down
E9Q6M5	Slc22a26	1.0775	0.0090	Down
Q00898	Serpina1e	2.7959	0.0016	Up
A0A0R4J135	Selenbp2	3.3062	0.0051	Up
Q9D1M7	Fkbp11	1.0331	0.0061	Up
P06683	C9	1.0150	0.0074	Up
Q61024	Asns	3.3282	0.0105	Up
E9Q6D8	C6	1.2909	0.0137	Up

KEGG enrichment analysis revealed that TLR4 deletion altered multiple pathways enriched in hepatic inflammation, metabolic regulation, inflammatory mediator-regulated TRP channels, and hepatocellular carcinoma (**Figure 6C**). Consistently, GO enrichment analysis demonstrated significant enrichment of functional categories related to chronic inflammatory response, regulation of IL-1β production, membrane organization, oxidoreductase activity, antigen binding, and metabolic processes (**Figure 6D**). These findings suggest that TLR4 contributes not only to inflammatory signaling but also to metabolic remodeling during *E. multilocularis*-induced hepatic injury.

### 3.7 TLR4 deficiency suppresses proliferative remodeling and reduces hepatic GHSR expression

**Figure 7:**
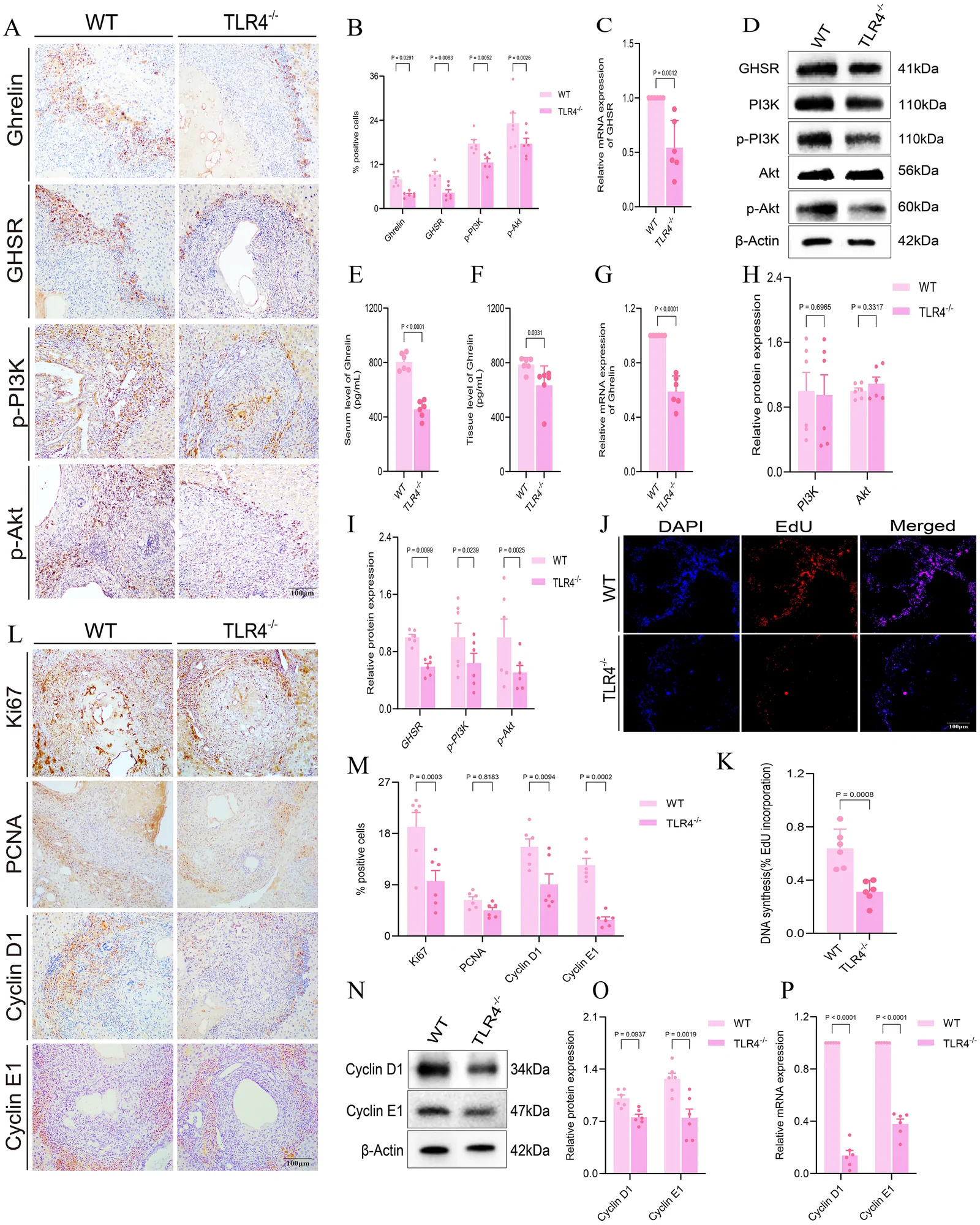
TLR4 deficiency suppresses proliferative remodeling and reduces hepatic GHSR expression. **(A, B)** Immunohistochemical staining and positive-cell quantification for Ghrelin, GHSR, p-PI3K and p-Akt in paraffin-embedded liver slices **(C)** qRT-PCR quantification of hepatic Ghsr transcript abundance. **(D, H, I)** Immunoblot profiling and densitometric statistics of GHSR, total PI3K/Akt and phosphorylated p-PI3K/p-Akt; β-Actin served as endogenous loading control. **(E, F)** ELISA-based detection of Ghrelin content within peripheral serum and hepatic homogenate, respectively. **(G)** qPCR measurement of relative hepatic Ghrelin mRNA expression. **(L, M)** IHC staining and positive-cell counting of proliferative markers Ki67, PCNA, Cyclin D1 and Cyclin E1. **(J, K)** Representative EdU fluorescence images and statistical calculation of peri-lesional proliferative cell percentage. **(N, O)** Western blot detection and quantitative analysis of Cyclin D1 and Cyclin E1 protein abundance. **(P)** qRT-PCR analysis of hepatic Cyclin D1 and Cyclin E1 mRNA levels. (n = 6). Statistical significance: *P < 0.05, **P < 0.01, ***P < 0.001, ****P < 0.0001.

This study further investigated the impact of TLR4 deficiency on hepatic proliferative remodeling during *E. multilocularis* infection. IHC analysis demonstrated reduced expression of Ghrelin, GHSR, p-PI3K, and p-Akt within perilesional inflammatory regions of TLR4-/- mice relative to WT controls, accompanied by decreased hepatic GHSR mRNA expression (**Figures 7A–C**). Consistent with these findings, Western blot analysis confirmed lower hepatic protein levels of GHSR, p-PI3K, and p-Akt in TLR4-deficient mice, while total PI3K and Akt protein expression remained unchanged (**Figures 7D, H, I**). In addition, both hepatic and circulating Ghrelin levels were reduced following TLR4 deletion (**Figures 7E–G**).

EdU staining further revealed a lower proportion of proliferating hepatocytes in perilesional hepatic tissues of TLR4-/- mice (**Figures 7J, K**). Consistently, IHC, Western blotting, and qRT-PCR analyses demonstrated reduced expression of key proliferation-associated markers, including Ki67, PCNA, Cyclin D1, and Cyclin E1, in the perilesional regions of TLR4-deficient livers (**Figures 7L–P**). Collectively, these findings indicate that TLR4 deficiency suppresses activation of the Ghrelin–GHSR/PI3K–Akt signaling cascade and attenuates aberrant perilesional proliferative remodeling during *E. multilocularis* infection. The coordinated downregulation of GHSR-associated signaling molecules and proliferative markers indicates that TLR4 and GHSR are jointly engaged in modulating hepatic reparative remodeling during AE pathogenesis.

### 3.8 TLR4 deficiency restrains hepatic inflammation and restores immune homeostasis

**Figure 8:**
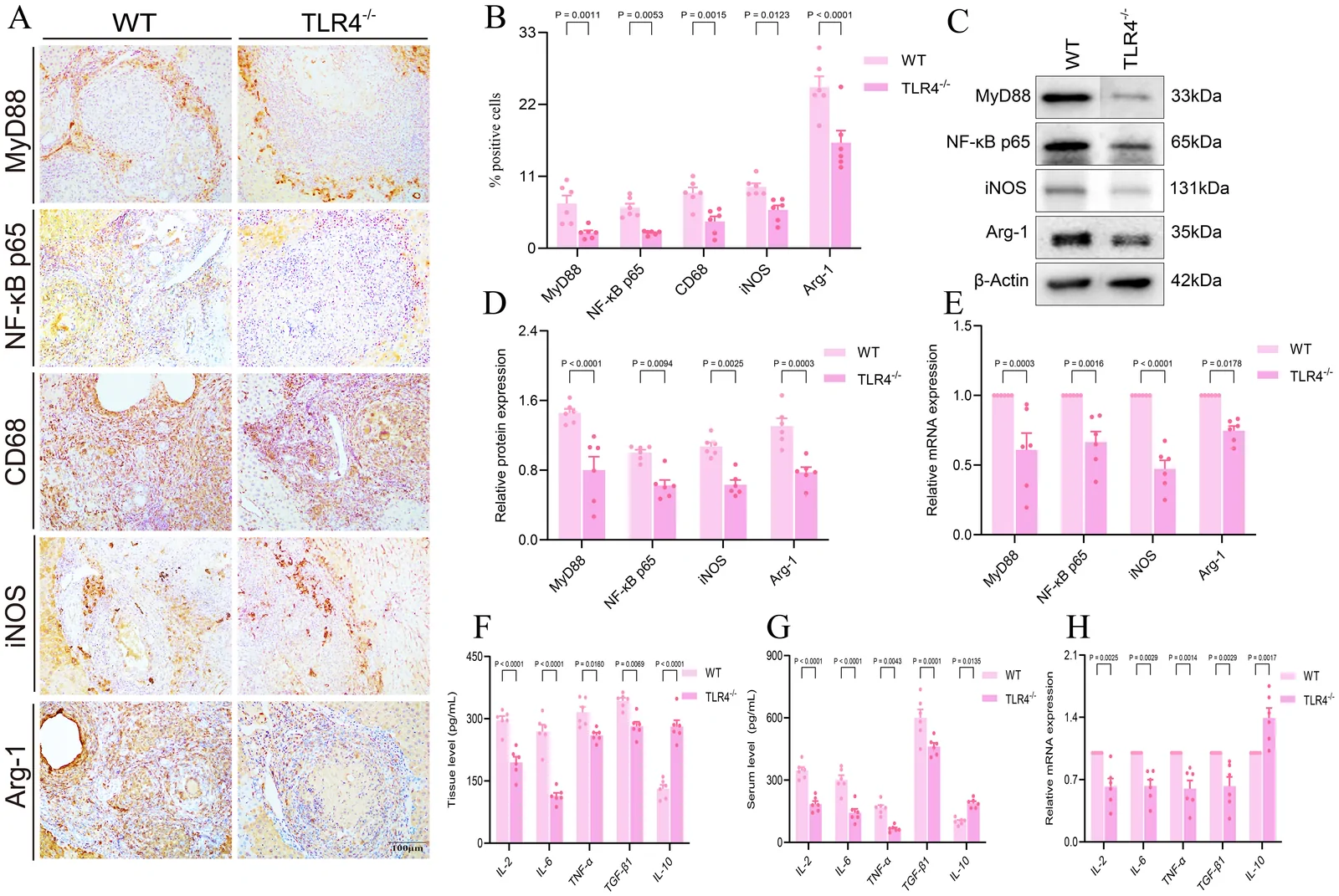
TLR4 deficiency restrains hepatic inflammation and restores immune homeostasis. **(A, B)** Immunohistochemical staining and quantitative counting of MyD88, NF-κB p65, CD68, iNOS and Arg- 1 positive cells in paraffin hepatic slices. **(C, D)** Immunoblot detection and densitometric quantification for MyD88, NF-κB p65, iNOS and Arg- 1; β-Actin was used as internal loading reference. **(E)** qRT-PCR measurement of hepatic mRNA expression levels of the above target genes. **(F, G)** ELISA quantification of five inflammatory cytokines (IL-2, IL-6, TNF-α, TGF-β1, IL- 10) in liver homogenate and peripheral serum respectively. **(H)** qRT-PCR analysis for relative cytokine mRNA abundance in liver tissue. (n = 6). Statistical significance: *P < 0.05, **P < 0.01, ***P < 0.001, ****P < 0.0001.

This study further investigated the effects of TLR4 deletion on the hepatic immune microenvironment during *E. multilocularis* infection. Quantitative IHC analysis was performed to evaluate the expression and spatial distribution of key inflammatory mediators and macrophage-associated markers in perilesional inflammatory regions. The protein levels of MyD88, NF-κB p65, CD68, iNOS, and Arg-1 were significantly lower in the TLR4-/- group relative to the WT group. Spatially, these molecules were predominantly localized to the peripheral areas of inflammatory lesions, whereas Arg-1 was mainly enriched within the central inflammatory regions (**Figures 8A, B**).

Western blotting and qRT-PCR further validated these histological observations. Both protein and mRNA expression levels of MyD88, NF-κB p65, iNOS, and Arg-1 were reduced in the livers of TLR4-/- mice compared with WT controls (**Figures 8C–E**). Notably, Arg-1 expression remained relatively higher than iNOS expression across all experimental groups, suggesting dominant M2 macrophage polarization during the late stage of infection.

To further characterize inflammatory responses, ELISA and qRT-PCR were performed to detect cytokine profiles in liver tissues and serum samples. TLR4 deficiency significantly reduced the levels of pro-inflammatory cytokines, including IL-2, IL-6, TNF-α, and TGF-β1, while elevating the level of the anti-inflammatory cytokine IL-10 (**Figures 8F–H**). Collectively, these findings demonstrate that TLR4 deficiency suppresses NF-κB-mediated inflammatory signaling and remodels the hepatic immune microenvironment during *E. multilocularis* infection. Although M2-associated macrophage features were observed across all groups, deletion of TLR4 reduced macrophage infiltration and inflammatory cytokine production while potentiating anti-inflammatory responses. These alterations facilitate the restoration of hepatic immune homeostasis and the attenuation of chronic inflammatory liver injury during AE progression.

## 4 Discussion

The ghrelin/GHSR signaling axis is a pivotal regulatory module that modulates somatic growth, hepatic metabolism, and immune homeostasis, and is involved in the pathological progression of multiple chronic liver diseases. Previous studies have demonstrated that ghrelin ameliorates hepatic injury by inhibiting NF-κB signaling activation and modulating autophagic flux (45). Nevertheless, genetic ablation of GHSR mitigates hepatic fibrosis and inflammatory infiltration in carbon tetrachloride (CCl_4_)-induced liver injury models (15). Earlier studies also revealed elevated hepatic ghrelin expression during *Echinococcus granulosus* (*E. granulosus*) infection, which facilitates disease progression via negative modulation of the TLR4/NF-κB inflammatory cascade (46), consistent with our previous findings (43). Our subsequent work further indicated that GHSR deficiency reduces endogenous ghrelin abundance, alleviates hepatic inflammatory responses, and ameliorates pathological liver damage during *E. granulosus* infection (47). In line with these preliminary findings, the present study systematically explored the individual and coordinated roles of GHSR and TLR4 in *E. multilocularis*-induced AE. Our phenotypic data showed that genetic deletion of either GHSR or TLR4 reduced hepatic lesion area, collagen deposition, glycogen metabolic disorder, and hepatic functional impairment during AE infection, accompanied by decreased hepatic and circulating ghrelin levels. These observations indicate that the ghrelin/GHSR axis exerts dual regulatory functions on hepatic metabolic reprogramming and immune responses throughout *E. multilocularis* infection.

Mechanistically, the protective effects associated with GHSR and TLR4 deficiency are closely associated with the suppression of ghrelin-mediated signaling. Although ghrelin exerts anti-inflammatory properties in several liver injury settings, sustained activation of the ghrelin–GHSR axis during chronic parasitic infection may simultaneously drive pathological hepatic remodeling. Excessive perilesional proliferative responses reshape the hepatic microenvironment to favor parasite persistence and long-term colonization, thereby accelerating AE progression. In the present study, parallel knockout models revealed coordinated expression patterns between GHSR and TLR4. GHSR deficiency was accompanied by downregulated TLR4 expression, whereas TLR4 deletion diminished hepatic GHSR expression and ghrelin abundance. These observations suggest that GHSR and TLR4 operate within a coordinated regulatory network that sustains inflammatory and proliferative responses during infection. Disruption of either component attenuated hepatic proliferative remodeling and chronic inflammatory activation, ultimately contributing to reduced liver injury.

Hepatic proliferative repair serves as a fundamental host defense mechanism against parasitic infection. However, uncontrolled compensatory proliferation contributes to the formation of a nutrient-rich microenvironment that supports parasitic growth and invasion (48). Multiple studies on hepatic echinococcosis have reported that suppression of perilesional cellular proliferation restricts parasitic development and improves disease outcomes (49–54). Consistent with these reports, our results demonstrated that *E. multilocularis* infection activates the PI3K/Akt signaling cascade and upregulates the expression of proliferation-related markers, including Ki67, PCNA, Cyclin D1, and Cyclin E1, thereby enhancing perilesional proliferative capacity. In contrast, both GHSR and TLR4 ablation blunted PI3K/Akt signaling activation and reversed the pro-proliferative molecular profile. Notably, the present study mainly focused on host hepatic inflammatory responses and aberrant proliferative remodeling, and direct detection of parasite viability or activity was not performed *in vivo*. Although our previous *in vitro* study has validated that GHSR inhibition impairs PSC survival and structural integrity, confirming the regulatory role of ghrelin/GHSR signaling in parasite activity (46). the association between host hepatic microenvironmental alterations and parasite progression in AE remains speculative. The present findings support a potential hypothesis that GHSR or TLR4 deficiency-induced microenvironmental changes may restrict parasite growth and delay AE progression, while definitive validation requires further dedicated experimental verification.

Inflammatory responses play central regulatory roles in the course of *E. multilocularis* infection (55). As core innate immune effector cells, hepatic macrophages dominate local microenvironmental remodeling and host defense against parasitic infection. Inhibition of GHSR (13–15, 21) and TLR4 signaling cascades (56–58) suppresses the downstream MyD88/NF-κB pathway, modulates macrophage phenotypic polarization, and reduces pro-inflammatory cytokine secretion, thereby relieving hepatic inflammatory injury. In the *E. multilocularis* infection model, predominantly monocyte-derived macrophages in the hepatic microenvironment mainly exhibit an iNOS+ pro-inflammatory phenotype in the early stage and gradually shift to an Arg-1+ anti-inflammatory phenotype in the late stage (59, 60). In the present study, GHSR or TLR4 deletion restrained TLR4/NF-κB signaling activation, reduced the infiltration of both M1 and M2 macrophage subsets, decreased pro-inflammatory cytokine levels (IL-2, IL-6, TNF-α, TGF-β1), and elevated anti-inflammatory IL-10 levels. Despite the dominant M2 macrophage phenotype in late-stage AE tissues, pro-inflammatory mediators remained prevalent in WT mice, sustaining persistent hepatic inflammation. The host initiates proliferative repair to compensate for infection-induced tissue damage, but this reparative process concurrently provides nutritional support for parasites, forming a self-amplifying “inflammation–proliferation–nutrient supply” feed-forward loop that exacerbates hepatic injury. However, GHSR ablation or TLR4-mediated indirect GHSR inhibition disrupts this pathological cycle. Impaired proliferative repair reduces local nutrient availability for parasites, while suppressed inflammatory signaling alleviates persistent hepatic immune-mediated hepatic damage. Therefore, GHSR functions as a core regulatory hub that bridges TLR4/NF-κB-dependent inflammation and aberrant proliferative repair during AE pathogenesis.

Proteomic profiling further corroborates the coordinated involvement of GHSR and TLR4 in AE pathogenesis. Differentially expressed proteins identified following GHSR or TLR4 deletion were enriched in pathways associated with inflammatory regulation, PI3K/Akt signaling, macrophage function, immune responses, and metabolic processes. These findings extend the histological and molecular observations by providing systematic *in vivo* evidence that GHSR and TLR4 participate in overlapping regulatory networks governing hepatic inflammatory and reparative networks. The consistent results across proteomic, histological, and biochemical data suggests that GHSR- and TLR4-dependent responses are not independent events, but core components of a coordinated pathogenic program that interconnects chronic inflammation, metabolic adaptation, and aberrant tissue remodeling during AE progression.

**Figure 9:**
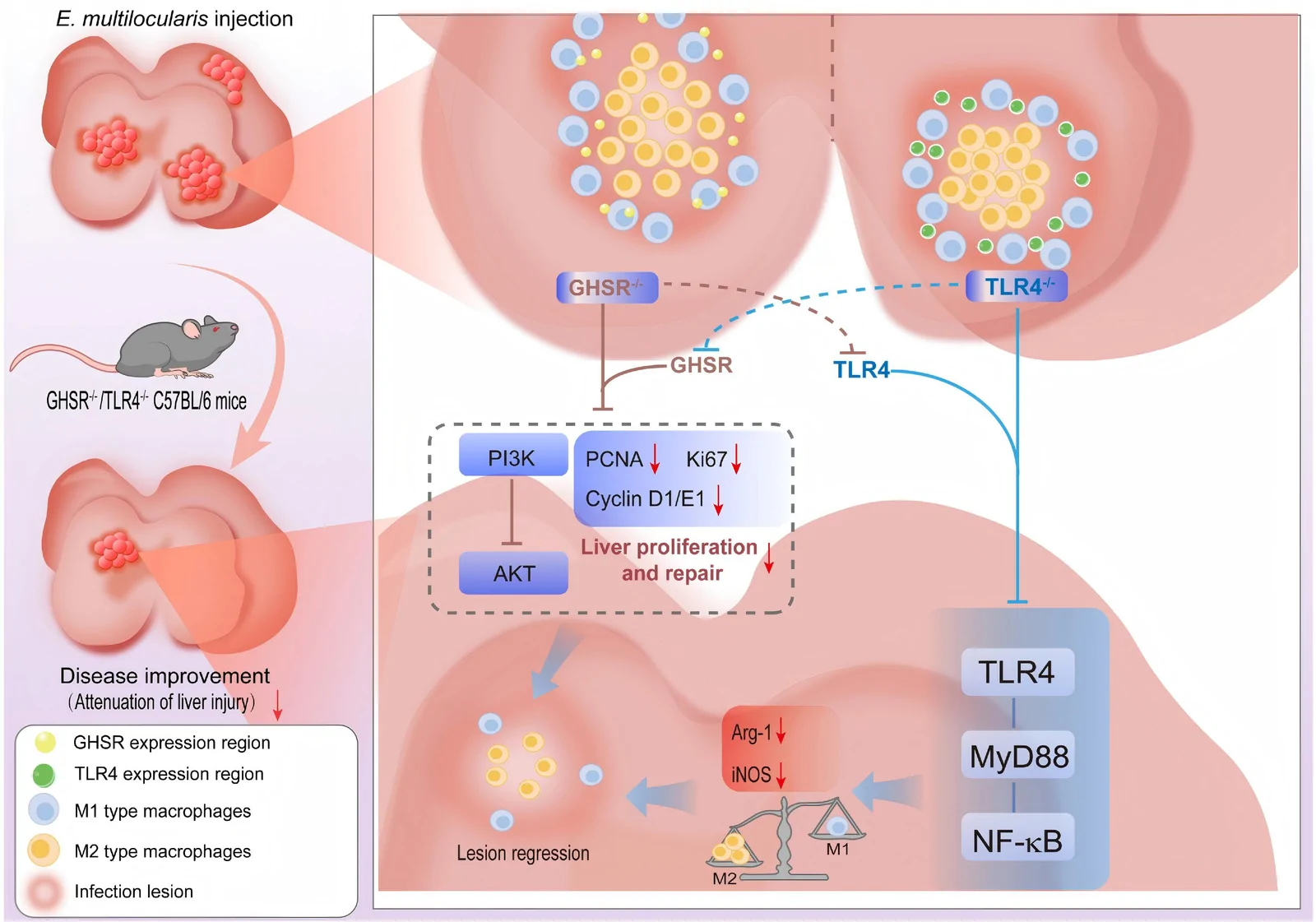
GHSR and TLR4 collectively promote hepatic inflammation and aberrant repair in alveolar echinococcosis. Genetic ablation of GHSR confers hepatic protection against *E.multilocularis*-induced damage via two coordinated pathways (1): dampening TLR4/MyD88/NF-ĸB cascade activity, which limits perilesional macrophage infiltration and reduces the secretion of pro-inflammatory mediators (2); inhibiting ghrelin-dependent PI3K/Akt signaling to restrain aberrant compensatory proliferative repair around parasitic lesions. These two biological alterations jointly slow focal lesion expansion and alleviate hepatic pathological injury. Meanwhile, TLR4 deletion achieves comparable hepatoprotective effects through interconnected regulatory routes: on one hand, suppressed MyD88/NF-ĸB signaling curbs macrophage recruitment and inflammatory cytokine production within lesion-surrounding tissues; on the other hand, TLR4 deficiency downregulates endogenous GHSR expression and subsequent ghrelin synthesis, indirectly blunting PI3K/Akt-driven proliferative responses. Collectively, knockout of either receptor interrupts the pathological inflammation-proliferation loop and relieves *E.multilocularis*-triggered liver damage through complementary modulation of inflammatory cascades and reparative proliferation.

In summary, the present study elucidates the cooperative pro-proliferative and pro-inflammatory functions of GHSR and TLR4, as well as their coordinated functional correlation during *E. multilocularis* infection (**Figure 9**). GHSR deficiency impairs infection-induced hepatic proliferative repair and alleviates chronic inflammatory injury by suppressing TLR4/NF-κB signaling cascade, thereby improving AE-associated hepatic pathological damage. Nevertheless, several limitations of this study should be acknowledged. The precise molecular interaction mechanism between GHSR and TLR4 remains to be fully clarified, and the potential synergistic or hierarchical regulatory patterns of the two receptors require further validation. First, this study only verifies the functional crosstalk between GHSR and TLR4 at the tissue and molecular levels *in vivo*. Molecular docking analysis provides preliminary in silico evidence of a potential structural association; however, direct physical or biochemical interaction between these two receptors has not been confirmed. The precise molecular regulatory relationship between GHSR and TLR4 requires further in-depth validation. Second, the present study focuses primarily on host hepatic inflammatory and reparative responses, and direct detection of parasite viability and activity was not performed. Thus, the causal relationship between altered host hepatic microenvironment and parasite survival remains speculative, which needs to be further verified by specialized parasite activity assays in future studies. In addition, the cell-type-specific functions of GHSR and TLR4 in hepatocytes, macrophages, and other hepatic resident cells remain to be systematically explored. Collectively, this study provides systematic *in vivo* phenotypic and proteomic evidence for the cooperative pro-pathological roles of GHSR and TLR4, offering novel mechanistic insights and potential therapeutic targets for the treatment of parasitic liver diseases.

## Data Availability

The raw mass‑spectrometry proteomics data have been deposited to the iProX database (ProteomeXchange consortium) under accession number IPX0019297000.
