# Supplementary material for: GHSR and TLR4 collectively promote hepatic inflammation and aberrant repair in alveolar echinococcosis
Source: Front Immunol. 2026 Sep 8;17:1919853. doi: 10.3389/fimmu.2026.1919853 (PMC13593426; doi:10.3389/fimmu.2026.1919853)
Supplement: Supplementary file 1 [file DataSheet1.zip › WB whole-membrane plots/GHSR/Order of Protein Sample Loading.doc]

**Order of Protein Sample Loading**

WT（3） GHSR^-/-^（3）
